# Clinical Characteristics of Inpatients with Childhood vs. Adolescent Anorexia Nervosa

**DOI:** 10.3390/nu11112593

**Published:** 2019-10-28

**Authors:** Charlotte Jaite, Katharina Bühren, Brigitte Dahmen, Astrid Dempfle, Katja Becker, Christoph U. Correll, Karin M. Egberts, Stefan Ehrlich, Christian Fleischhaker, Alexander von Gontard, Freia Hahn, David Kolar, Michael Kaess, Tanja Legenbauer, Tobias J. Renner, Ulrike Schulze, Judith Sinzig, Ellen Thomae, Linda Weber, Ida Wessing, Gisela Antony, Johannes Hebebrand, Manuel Föcker, Beate Herpertz-Dahlmann

**Affiliations:** 1 Department of Child and Adolescent Psychiatry, Psychosomatics and Psychotherapy, Charité–Universitätsmedizin Berlin, corporate member of Freie Universität Berlin, Humboldt-Universität zu Berlin, and Berlin Institute of Health, 13353 Berlin, Germany; 2 Department of Child and Adolescent Psychiatry, Psychosomatics and Psychotherapy, University Hospital, RWTH Aachen, 52074 Aachen, Germany; 3 Institute of Medical Informatics and Statistics, Kiel University, 24105 Kiel, Germany; 4 Department of Child and Adolescent Psychiatry, Psychosomatics and Psychotherapy, Philipps-University Marburg and University Hospital Marburg, 35039 Marburg, Germany; 5 Center for Mind, Brain and Behavior (CMBB), Philipps-University Marburg, 35032 Marburg, Germany; 6 The Zucker Hillside Hospital, Department of Psychiatry, Northwell Health, Glen Oaks, NY 11004, USA; 7 Hofstra Northwell School of Medicine, Department of Psychiatry and Molecular Medicine, Hempstead, NY 11549, USA; 8 Department of Child and Adolescent Psychiatry, Psychosomatics and Psychotherapy, University Hospital Wuerzburg, 97080 Wuerzburg, Germany; 9 Department of Child and Adolescent Psychiatry & Division of Psychological & Social Medicine and Developmental Neurosciences, Faculty of Medicine, Technische Universität Dresden, 01307 Dresden, Germany; 10 Department of Child and Adolescent Psychiatry and Psychotherapy, University Medical Center Freiburg, 79104 Freiburg, Germany; 11 Department of Child and Adolescent Psychiatry, Saarland University Hospital, 66421 Homburg, Germany; 12 Department of Child and Adolescent Psychiatry, Psychosomatics and Psychotherapy, LVR–Hospital Viersen, 41749 Viersen, Germany; 13 Department of Child and Adolescent Psychiatry, University Medicine of the Johannes Gutenberg-University, 55131 Mainz, Germany; 14 Clinic of Child and Adolescent Psychiatry, Center for Psychosocial Medicine, University Hospital Heidelberg, 69115 Heidelberg, Germany; 15 University Hospital of Child and Adolescent Psychiatry and Psychotherapy, University of Bern, 3000 Bern, Switzerland; 16 LWL University Hospital Hamm for Child and Adolescent Psychiatry, Ruhr University Bochum, 59071 Hamm, Germany; 17 Department of Child and Adolescent Psychiatry, Psychosomatics and Psychotherapy, University Hospital Tuebingen, 72076 Tuebingen, Germany; 18 Department of Child and Adolescent Psychiatry/Psychotherapy, University Hospital, University of Ulm, 89075 Ulm, Germany; 19 Department of Child and Adolescent Psychiatry, Psychosomatics and Psychotherapy, LVR-Klinik Bonn, 53111 Bonn, Germany; 20 Department of Child and Adolescent Psychiatry, Faculty of Medicine, Technische Universität Dresden, 01307 Dresden, Germany; 21 Department of Child and Adolescent Psychiatry, Psychosomatics and Psychotherapy, University Hospital Muenster, 48149 Muenster, Germany; 22 Central Information Office KKNMS, Philipps-University Marburg, 35112 Bellnhausen, Germany; 23 Department of Child and Adolescent Psychiatry, Psychotherapy, and Psychosomatics, University Hospital Essen, University of Duisburg-Essen, 45147 Essen, Germany

**Keywords:** anorexia nervosa, children, adolescents, clinical characteristics, BMI, outcome

## Abstract

We aimed to compare the clinical data at first presentation to inpatient treatment of children (<14 years) vs. adolescents (≥14 years) with anorexia nervosa (AN), focusing on duration of illness before hospital admission and body mass index (BMI) at admission and discharge, proven predictors of the outcomes of adolescent AN. Clinical data at first admission and at discharge in 289 inpatients with AN (children: *n* = 72; adolescents: *n* = 217) from a German multicenter, web-based registry for consecutively enrolled patients with childhood and adolescent AN were analyzed. Inclusion criteria were a maximum age of 18 years, first inpatient treatment due to AN, and a BMI <10th BMI percentile at admission. Compared to adolescents, children with AN had a shorter duration of illness before admission (median: 6.0 months vs. 8.0 months, *p* = 0.004) and higher BMI percentiles at admission (median: 0.7 vs. 0.2, *p* = 0.004) as well as at discharge (median: 19.3 vs. 15.1, *p* = 0.011). Thus, in our study, children with AN exhibited clinical characteristics that have been associated with better outcomes, including higher admission and discharge BMI percentile. Future studies should examine whether these factors are actually associated with positive long-term outcomes in children.

## 1. Introduction

Anorexia nervosa (AN) is one of the most severe psychiatric disorders in children and adolescents and has substantial morbidity and mortality [1]. The peak age of onset for AN is 15 to 17 years [2]; but there is some evidence that the age of onset has decreased over the last decades [3,4,5,6]. Increasing hospital admissions of patients with AN <15 years have been detected in Germany [7] and the UK [8]. However, knowledge of the clinical features at admission to hospital treatment in children with AN remains limited (for an overview please see [9]). To date, only few studies have examined differences in the features of AN in children vs. in adolescents, yielding inconsistent results. For example, Peebles et al. [10] compared clinical characteristics of children (age at presentation <13 years, mean age: 11.6 years) and adolescents (age at presentation 13–20 years, mean age: 15.6 years) with an eating disorder (ED) diagnosis in a large sample of 959 in- and outpatients via retrospective record review. The authors reported that younger AN patients presented with a lower percentage of ideal body weight, had lost weight more rapidly, and showed a shorter illness duration than adolescents. A very recent study in an Asian population of AN patients [11] compared patients with childhood-onset (mean age of onset 11.5 years) and adolescent-onset (mean age of onset 15.2 years). Different from the prior study, the authors found in the childhood-onset group a longer duration of illness before inpatient treatment (4.8 vs. 2.6 years) and a longer duration of hospital stay (6.0 vs. 3.2 weeks). This is in line with preliminary results from a German study [12] that included 40 patients with childhood-onset AN (<13 years) and 53 patients with adolescent-onset (13–18 years). The results of this study indicate that childhood-onset AN is associated with a longer duration of untreated illness (mean: 38.4 months) than adolescent-onset AN (mean: 20.8 months) and adult-onset AN (mean: 19.0 months). A prospective, long-term outcome study by Wentz et al. [13], which included adolescents as well as a small sample of children with AN, showed that childhood-onset AN predicted a longer duration of the first AN episode.

General practitioners and pediatricians play an important role in the initial diagnosis of AN [12]. A less typical presentation of AN symptoms in childhood might delay the diagnosis and treatment initiation [14]. However, the duration of illness and body mass index (BMI) at admission and discharge have emerged as significant predictive factors for the outcome of AN. Several early follow-up studies in children with AN demonstrated a worse outcome than in adolescents (for an overview see [15]), which was confirmed in a recent study of AN patients with an onset age <14 years [15] and a recent 30-year follow-up study [16].

Thus, the aim of this study was to determine whether clinical parameters, such as premorbid BMI percentile, rate of weight loss before admission, duration of illness, outpatient contacts before admission, psychiatric comorbidities, blood parameter measuring starvation at admission (fT3) and BMI percentile at admission and discharge differed in a large sample of children and adolescents with AN.

## 2. Materials and Methods

### 2.1. Study Design and Participants

This study was based on data from the ongoing German web-based registry for inpatients with childhood and adolescent AN (Kompetenznetz Anorexie-Register Deutschland e.V.), with 14 participating German university hospital departments of child and adolescent psychiatry as well as two major non-university hospitals taking part in this multicenter study [14]. The registry was established in January 2015 to systematically collect AN-specific healthcare data. The inclusion criteria for the present analysis included children and adolescents up to the age of ≤18 years diagnosed with AN (according to DSM-5 criteria) by a child and adolescent psychiatrist and first admission to inpatient treatment because of AN. The weight threshold criterion was a BMI <10th percentile (in accordance with international definitions of adolescent AN) [17,18,19]. Included patients were admitted consecutively to first inpatient treatment due to AN between January 2015 and March 2019. All patients received a multimodal, multidisciplinary cognitive behavioral therapy (CBT)-based and family-oriented inpatient treatment program that included weight restoration, nutritional counselling, individual and group therapy, group psychoeducation program for parents and family sessions [20]. If indicated, patients were also treated with psychotropic medications.

Our definitions of childhood AN (<14 years at admission to hospital treatment) and adolescent AN (≥14 years at admission to hospital treatment) correspond to those reported in previous studies on childhood and adolescent AN from our group as well as with the legal German age definition of childhood and adolescence.

Written informed consent was obtained from all patients and their legal guardians. The study was approved by the ethics committees of all participating study sites (see Supplement Table S1). The methodological standards of the registry for data collection corresponded to Good Clinical Practice (GCP) regulations.

### 2.2. Assessments

The following clinical data at admission and at discharge of children <14 years and adolescents ≥14 years were collected: sex, premorbid weight (kg) and height (cm), weight loss (kg) per month prior to admission, weight (kg) and height (cm) at admission and at discharge, clinically derived diagnosis of AN subtype and of psychiatric comorbidity, blood parameter measuring starvation at admission (fT3), and duration of hospital treatment (in weeks). Moreover, patients and their parents specified the duration of illness before admission (in months), i.e., the elapsed time between the initiation of weight loss or insufficient age-appropriate weight gain and admission as well as the number of outpatient consultations because of ED symptoms before admission to hospital.

Age- and sex-adjusted BMI percentiles were calculated using normative data from the KIGGS study, a large German reference population [21], as in childhood and adolescence, age-adapted BMI percentiles or expected body weight (EBW) values should be used because body weight and height change non-linearly during pubertal development [17,22,23].

Moreover, we assessed the following sociodemographic parameters: the living situation of the patient (i.e., living with both parents or single parent), the number of siblings, the type of school attended by the patient at the time of admission and data on the parental educational attainment.

### 2.3. Statistical Analysis

For descriptive statistics of continuous variables, means (M) and standard deviations (SD) (for symmetric distributions) or median and quartiles (for skewed distributions) were calculated. For nominal variables, numbers and percentages are presented. Differences in continuous variables between children and adolescents with AN were evaluated using Mann–Whitney U Test and Chi-Squared Test for nominal variables. Values of *p* < 0.05 were considered statistically significant. All statistical analyses were two-sided and performed using the Statistical Package for Social Sciences Version 22 [24].

## 3. Results

### 3.1. Sample Characteristics

Between January 2015 and March 2019, 289 patients (*n* = 72 (24.9%) children aged 8–13 years and *n* = 217 (75.1%) adolescents aged 14–18 years) were included in the registry who were consecutively admitted to first inpatient treatment due to AN.

### 3.2. Clinical Characteristics at Admission and at Discharge

The mean age of onset was 12.4 years in children and 15.0 years in adolescents. Compared to adolescents with AN, children with AN had a shorter median illness duration before hospitalization. Age at admission and duration of illness correlated positively (*p* < 0.001). Groups did not differ in mean premorbid BMI percentiles, mean rate of weight loss before admission as well as the proportion of patients who had at least two outpatient contacts before admission.

The median age at admission was 13.2 years in the childhood AN sample and 15.6 years in the adolescent AN sample. In the childhood AN sample, 36% of the patients were <13 years old at admission (see Supplement Table S2). There were no significant differences in the sex ratio and the rate of psychiatric comorbidities between both groups. With an average body weight of 45.3 ± 8.8 kg in children in comparison to 55.8 ± 7.5 kg adolescents at the onset of illness, weight loss amounted to an average of 20.3% of total body weight in children in contrast to an average of 22.5% of total body weight in adolescents (*p* < 0.05).

The prevalence of AN subtypes differed significantly between children and adolescents, with the restricting subtype being predominant in both groups, but with fewer children than adolescents fulfilling the AN binge-purging subtype. In children, fT3 was significantly less often within the normal range compared to adolescents. The median age- and sex-adjusted BMI percentiles at admission as well as at discharge were significantly higher in children than in adolescents.

The two age groups did not significantly differ in the median duration of hospital treatment (see Table 1 and Table 2).

### 3.3. Sociodemographic Parameters at Admission

At admission, children and adolescents with AN did not differ significantly in the sociodemographic variables described above (parental situation, number of siblings, school type and parental educational attainment) (see Supplement Table S3).

## 4. Discussion

The aim of this study was to compare the clinical presentation of children vs. adolescents admitted to a first hospital treatment because of AN. There was a focus on the parameters of duration of illness before admission [25,26,27] and BMI at admission [25,26] and discharge [28], as those parameters have been shown previously to be significant predictive factors for the outcomes of adolescent AN [28,29,30]. In addition, we also examined factors that possibly influence treatment outcomes, such as premorbid BMI, rate of weight loss, outpatient treatment before admission, psychiatric comorbidity and blood parameter of starvation (fT3) at admission. To our knowledge, our results are based on one of the largest recent samples of children and adolescents with AN from a multicenter study.

In our study, children with AN (mean age of onset 12 years) had a shorter illness duration before admission to inpatient treatment and a higher BMI percentile at admission than adolescents. In contrast to our results Walker et al. [31] did not find significant differences in mean BMI z-score between children (≤12 years) and adolescents (13–18 years) with EDs at referral to a specialist pediatric program. The mean duration of illness is comparable with that of our study, but Walker et al. also included patients with bulimia nervosa and with other or unspecified EDs.

Furthermore, our findings are in line with the results of the British National Surveillance Study [4], which indicated a similar length of illness prior to treatment (8.3 months) in patients with early-onset EDs (≤14 years). However, our findings are inconsistent with the results of a German study [12], in which childhood-onset AN was associated with the longest duration of untreated illness compared to adolescent-onset and adult AN. These divergent results might be due to different samples in both studies. We investigated patients who were treated at major hospitals in inpatient settings, whereas the other study recruited patients in different treatment settings, e.g., inpatient units, day clinics, outpatient departments and ED-specific counselling centers. Thus, our sample was much more homogeneous than that of Neubauer et al. [12]. Moreover, the sample of Neubauer et al. [12] was restricted to a certain region in Germany, which might have influenced diagnostic procedures and allocation criteria. In contrast to our study, a very recent Asian study [11] found a significantly longer illness duration before inpatient treatment in patients with childhood-onset AN than in patients with adolescent-onset AN, although the age of onset was comparable in both studies. However, the duration of illness was much longer in the study of Kwok et al. [11] than in our study, not only in children (4.8 years vs. 6.4 months), but also in adolescents (2.6 years vs. 9.6 months). These differences may be determined by different health care systems and admission criteria for inpatient treatment. This finding highlights that characteristics such as illness duration (and possibly closely related BMI at admission) may not be intrinsic to childhood or adolescent AN, but rather highly influenced by national health care systems, in which different opportunities for early detection and treatment options are available and lead to inconsistent findings between studies in different countries. In Germany, inpatient treatment is the gold standard for the treatment of adolescent AN. In our sample, outpatient treatment before admission did not differ between children and adolescents.

Although children had a shorter duration of illness and displayed a similar rate of weight loss per month and a lower weight loss in percent of previous body weight compared to adolescents, a greater proportion of the childhood sample showed symptoms of acute starvation in terms of fT3 levels. The children had a higher body weight at admission although both groups showed no differences in premorbid weight. This finding might be due to the fact that parents of younger patients are more involved in their children’s everyday life who might have realized changes in dietary behaviour at an earlier stage than do parents of adolescent patients. Furthermore, in 1998, a regular health check-up for 12- to 15-year-olds (J1) was established in Germany. It covers a complete physical examination [32]. Therefore, the duration of untreated illness could be shortened in children vs. adolescents, and the extent of underweight at admission to hospital may have been less severe [33]. This is in contrast to previous studies conducted in other countries [11,31] demonstrating that children had a longer duration of illness [11] than adolescents or differed only slightly in the time period [31].

In our sample, children had a higher BMI percentile at discharge compared to adolescents, although the duration of inpatient treatment and number and type of psychiatric comorbidities, which also might influence treatment duration and outcome [34,35,36] did not differ between both groups. Föcker et al. [37] found that a higher BMI percentile at discharge was associated with a higher BMI at 1-year follow-up in a sample of 172 adolescents with first-onset AN.

Besides the investigation by Kwok et al. [11] conducted in Asia and thus not quite comparable to Western industrial states this is the first study to assess sociodemographic variables in children with AN versus adolescents. However, we could not find any significant difference between both age groups.

Taken together, according to our findings, children with AN seem to exhibit factors indicative of positive outcomes described in previous studies, such as higher body weight at admission and at discharge [15,28,30,37,38,39] and a shorter illness duration [29,40]. However, the presence of these factors cannot be directly taken as indicators of better long-term outcomes since multiple further factors determine medium- and long-term outcomes in AN, such as medical comorbidities [41], trauma [42] and family interaction [43,44].

### 4.1. Limitations

The findings of the present study must be interpreted in the context of several limitations. First, we only analyzed data from AN patients in need of hospital treatment. Hence, the studied patients probably had a more severe course of illness than outpatient or community-based samples to which our results may not generalize. Second, although the diagnosis was based on the evaluation of experienced child and adolescent clinicians, no structured clinical interview was conducted. Third, the assessment of clinical and psychological parameters was limited in this registry-based study, e.g., we had no data about probable risk factors such as a preceding trauma, accidents or mobbing. Moreover, no information regarding types of interventions before admission to inpatient treatment were available in the registry. Finally, we have no information beyond hospital discharge, e.g., on relapse and rehospitalization in the short or long-term. However, in a registry study with a large number of clinical investigators the assessment of reliable data is limited.

### 4.2. Strengths

Despite these limitations, the strengths of the current study are the relatively large sample size of patients included in a multicenter registry of juvenile AN patients that was not limited to a special region in Germany, and the considerably high number of children aged <14 years with AN. As far as we know this is one of the largest studies to compare clinical and sociodemographic data between children and adolescents admitted to hospital for the first time [11,31]. In addition, the prospective design of our study allows for a more definite interpretation of the results, in contrast to studies using a retrospective design [10,12]. The current study yields relevant information on differences in the clinical features of first-time hospitalized children and adolescents with AN.

## 5. Conclusions

In our study, children with AN exhibited several factors, such as a higher body weight at admission [25,26] and at discharge [28] and a shorter duration of illness [25,26,27], that have been observed to be relevant to positive outcomes in previous follow-up studies. However, previous studies have suggested that the long-term outcomes in childhood AN seem to be worse than those of adolescent AN. Evidently, positive outcomes at long-term follow-up are not only based on the achievement of a healthy body weight but also on changes in ED symptoms, motivation to recover, comorbidity and familial problems [25,45]. Moreover, risk factors such as a preceding trauma [27], teasing or mobbing by peers play an important role.

Thus, studies are needed that investigate a wide array of clinical, psychological, and social parameters in respect to outcome at discharge from hospitalization and beyond to improve treatment of this debilitating disorder in children with AN.

## Figures and Tables

**Table 1:** Clinical characteristics at admission.

	Children with AN *n* = 72		Adolescents with AN *n* = 217		Mann–Whitney U /X^2^-Test
	*n* (%)	M ± SD or Median (Q1, Q3)	*n* (%)	M ± SD or Median (Q1, Q3)	*p*
Age (in years) at admission		13.2 (12.1, 13.6)		15.6 (14.8, 16.6)	
Sex					0.821
Female	70 (97.2)		212 (97.7)		
Male	2 (2.8)		5 (2.3)		
AN subtype					0.014
restricting type	72 (100.0)		200 (92.2)		
binge-purging type	0 (0.0)		17 (7.8)		
Psychiatric comorbidity	22 (32.4)		87 (42.2)		0.156
Age of onset		12.4 ± 0.9		15.0 ± 1.1	
Duration of illness before admission (months)		6.0 (3.0, 8.0)		8.0 (5.0, 12.0)	0.004
At least two outpatient contacts before admission	44 (63.8)		140 (67.6)		0.559
Premorbid BMI		18.1 ± 2.3		20.3 ± 2.4	
Premorbid BMI percentile		37.5 ± 24.7		40.7 ± 24.8	0.384
Rate of weight loss before admission (in kg/month)		2.2 ± 2.4		2.2 ± 2.0	0.995
Height (cm)		158.1 ± 8.6		166.1 ± 6.9	
Height percentile		49.4 ± 28.6		54.2 ± 59.5	0.238
BMI		14.3 ± 1.3		15.5 ± 1.2	a
BMI percentile		0.7 (0.2, 2.3)		0.2 (0.0, 1.3)	0.004
fT3 within the normal range	18 (30.5)		86 (49.4)		0.015

BMI (kg/m^2^) = body mass index, fT3 = free triiodothyronine, Q1 = 1st quartile, Q3 = 3rd quartile. a: Differences between absolute BMI values of these two groups were not calculated because they are age- and height related and do not give any additional information about treatment outcome.

**Table 2:** Clinical characteristics at discharge.

	Children with AN *n* = 72	Adolescents with AN *n* = 217	Mann–Whitney U
	M ± SD or Median (Q1, Q3)	M ± SD or Median (Q1, Q3)	*p*
BMI at discharge	17.4 ± 1.4	18.6 ± 1.3	a
BMI percentile at discharge	19.3 (11.5, 27.3)	15.1 (7.0, 24.5)	0.011
Duration of hospital treatment (weeks)	17.0 (13.0, 21.0)	17.0 (12.0, 21.0)	0.771

BMI (kg/m^2^) = body mass index, Q1 = 1st quartile, Q3 = 3rd quartile. a: Differences between absolute BMI values of these two groups were not calculated because they are age- and height related and do not give any additional information about treatment outcome.

## Supplementary Materials

The following are available online at https://www.mdpi.com/2072-6643/11/11/2593/s1, Table S1: Ethics statement. List of all involved ethics committees; Table S2: Age, illness duration and sex distribution at admission of children and adolescents with AN; Table S3: Parental situation, number of siblings, school types of the patient and parental educational attainment.
