# Supplementary material for: Clinical Characteristics of Inpatients with Childhood vs. Adolescent Anorexia Nervosa
Source: Nutrients. 2019 Oct 28;11(11):2593. doi: 10.3390/nu11112593 (PMC6893829; doi:10.3390/nu11112593)
Supplement: Supplementary file 1 [file nutrients-11-02593-s001.pdf]

nutrients MDPI

Table S1. Ethics statement. List of all involved ethics committees.

#	Study center	Ethics committee	Reference number
1	Essen	ethics committee of the medical faculty of the University Essen	12-5169-BO
2	Aachen	ethics committee of the medical faculty of the RWTH Aachen	EK028/12
3	Mainz	ethics committee of the local medical advisory board Rheinland-Pfalz	837.130.16 (10449)
4	Marburg	ethics committee of the medical faculty of the Philipps-University Marburg	86/14
5	Würzburg	ethics committee of the medical faculty of the Julius-Maximilians-University Würzburg	180/13_z
6	Dresden	ethics committee of the TU Dresden	EK 342092014
7	Freiburg	ethics committee of the Albert-Ludwigs-University Freiburg	132/14
8	Viersen	ethics committee of the local medical advisory board Nordrhein	2014183
9	Homburg	ethics committee of the local medical advisory board Saarbrücken	220/13
10	Berlin	ethics committee (board nr. 2) of the Charité Berlin	EA2/003/14
11	Heidelberg	ethics committee of the medical faculty Heidelberg	S-244/2015
12	Hamm	ethics committee of the medical faculty of the Ruhr-Universität Bochum	5082-14
13	Tübingen	ethics committee of the medical faculty of the Eberhard-Karls-Universität Tübingen	343/2014BO1
14	Ulm	ethics committee of the university of Ulm	170/07
15	Bonn	joint vote with center in Viersen	
16	Münster	ethics committee of the medical advisory board Westfalen-Lippe and the Westfälische Wilhelms-Universität	2016-384-b-S

Table S2. Age, illness duration and sex distribution at admission of children and adolescents with AN.

	Age at admission (years)	Duration of illness before admission (months) M±SD	Females N=282 n (%)	Males N=7 n (%)
Children	8	a	1 (0.4)	0 (0)
	10	3.0±1.4	3 (1.1)	0 (0)
	11	6.6±3.0	10 (3.5)	0 (0)
	12	5.4±3.5	11 (3.9)	1 (14.3)
	13	7.8±4.9	45 (16.0)	1 (14.3)
Adolescents	14	8.0±4.9	64 (22.7)	0 (0)
	15	9.5±7.5	63 (22.3)	2 (28.6)
	16	10.3±6.9	51 (18.1)	2 (28.6)
	17	14.4±14.2	32 (11.3)	1 (14.3)
	18	22.0±0.0	2 (0.7)	0 (0)

a: Duration of illness was not calculated due to missing values.

Table S3. Parental situation, number of siblings, school types of the patient and parental educational attainment.

	Children with AN n=72	Adolescents with AN n=217	X^2-Test
	n (%)	n (%)	*p*
**Parental situation**	**71 (100)**	**212 (100)**	**0.77**
Living with both parents	51 (71.8)	161 (76.0)	
Living with one parent	20 (28.2)	51 (24.0)	
**Number of siblings**	**72 (100)**	**217 (100)**	**0.90**
0	11 (15.3)	30 (13.8)	
1	34 (47.2)	109 (50.2)	
≥2	27 (37.5)	78 (36.0)	
**School type***	**72 (100)**	**217 (100)**	**0.78**
„Gymnasium“	43 (59.7)	128 (59.0)	
„Gesamtschule“	9 (12.5)	24 (11.1)	
„Realschule“	14 (19.4)	41 (18.9)	
„Hauptschule“	3 (4.2)	6 (2.8)	
other secondary schools	3 (4.2)	18 (8.3)	
**Graduation father****	**61 (100)**	**189 (100)**	**0.39**
„Abitur“	37 (60.6)	116 (61.4)	
„Mittlere Reife“	14 (23.0)	51 (27.0)	
„Hauptschulabschluss“	10 (16.4)	18 (9.5)	
no graduation	0 (0)	2 (2.1)	
**Graduation mother****	**62 (100)**	**189 (100)**	**0.35**
„Abitur“	37 (59.7)	95 (50.3)	
„Mittlere Reife“	20 (32.3)	83 (43.9)	
„Hauptschulabschluss“	4 (6.5)	10 (5.3)	
no graduation	1 (1.6)	1 (0.5)	

*We decided to keep the German terms for the different school types and graduations, because they are not quite comparable to other countries` school systems. Gymnasium = school covering both the lower and upper secondary levels, in-depth general education aimed at the general higher education entrance qualification; Gesamtschule = type of school at the lower secondary level offering several grades of education leading to different qualifications; Realschule = school at the lower secondary level with extensive general education; Hauptschule = school at the lower secondary level with basic general education.

****Abitur** = Secondary school qualification after 12 or 13 years of school education, which constitutes the general higher education entrance qualifications; **Mittlere Reife** = general education school, which leaves a certificate obtained on completion of grade 10 at the Realschule; **Hauptschulabschluss** = general education school that leaves a certificate obtained on completion of grade 9 at the Hauptschule.
